# Antibiotic Resistance and Therapy for *Helicobacter pylori* Infection

**DOI:** 10.3390/antibiotics12121669

**Published:** 2023-11-28

**Authors:** Stella I. Smith, Yoshio Yamaoka

**Affiliations:** 1 Department of Molecular Biology and Biotechnology, Nigerian Institute of Medical Research, Yaba, Lagos 100001, Nigeria; 2 Department of Environmental and Preventive Medicine, Oita University Faculty of Medicine, Idaigaoka, Hasama-machi, Yufu 879-5593, Japan; yyamaoka@oita-u.ac.jp; 3 Department of Medicine-Gastroenterology and Hepatology Section, Baylor College of Medicine, Houston, TX 77030, USA

Approximately half of the world’s population is estimated to be infected with *Helicobacter pylori*. Patients infected with this pathogen typically develop chronic active gastritis, but peptic ulcer, mucosa-associated lymphoid tissue lymphoma, and gastric adenocarcinoma can ensue in a minority of cases. Antibiotic resistance, an increasingly serious threat to human health worldwide, is the major cause of treatment failure for *H. pylori* infection. Therefore, it is important to find new ways to improve/enhance treatment strategies to curb the increased antibiotic resistance of this bacterial pathogen and develop better management techniques. Several new strategies have been devised to study antibiotic resistance in *H. pylori*, with recent review articles of these methods proffering suggestions/solutions to properly manage and ultimately eradicate drug-resistant strains globally.

This Special Issue, entitled “*Antibiotic Resistance and Therapy for Helicobacter pylori Infection*”, highlights strategies for curbing antibiotic resistance in *H. pylori* and makes suggestions for the appropriate management of infections.

Fiorani et al. (contribution 1) discussed the influence of *H. pylori* on the human gastric and gut microbiota and their complex interactions. The authors suggested that eradication therapy used in the treatment of *H. pylori* could also have detrimental effects on the gut microbiota, decreasing its alpha diversity. Furthermore, they suggested the use of probiotic supplementation to not only improve cure rates, but also reduce side effects associated with antimicrobial usage and increase patient compliance.

To support the view of Fiorani et al. (contribution 1), Choudhry et al. [1] reported on the control of *H. pylori* with engineered selective guided antimicrobial peptides. The authors developed antimicrobial agents with high selectivity against *H. pylori*. They carried this out by fusing a *H. pylori*-binding guide peptide (MM1) to broad-spectrum antimicrobial peptides. They additionally engineered the *Lactococcus lactis* probiotic to secrete these peptides (gAMP). The gAMP probiotics were then tested in *H. pylori*-infected mice. In therapeutic terms, this method surpassed treatment with antibiotics, while successfully eliminating *H. pylori* in 5 days and simultaneously protecting the mice from challenge infection as a prophylactic.

Alfaray et al. (contribution 2) performed a global-scale study of *H. pylori* antimicrobial resistance genes, comparing various detection tools and analyzing antibiotic resistance genes (ARGs) and efflux pump genes, worldwide epidemiological distribution, and information related to the antimicrobial-resistant phenotype. They reported that *H. pylori* has 42 ARGs against 11 different antibiotic classes. Further research into these ARGs revealed that approximately 30% occur naturally in the core genome, whereas those in the accessory genome are restricted to particular strains. The 29 potential efflux pump genes identified were noted to be mostly located in the core genome. The authors finally concluded that having an ARG does not correspond to the resistant or sensitive phenotype of clinical isolates from patients, although the minimum inhibitory concentration may be influenced. 

Fiorini et al. [2] further conducted a study on antibiotic resistance of and therapy for *H. pylori* infection in immigrant patients treated in Italy. The authors concluded that, as a first-line treatment, Pylera^®^ attained a good performance for all naïve patients who were foreigners, with the exception of African migrants.

To further understand the critical resistance mechanisms in *H. pylori*, Fauzia et al. (contribution 3) conducted a regional study of mutations related to antibiotic resistance in this pathogen. Clinical isolates from Bangladeshi patients were used for whole-genome sequencing of the *pbp1a*, *rdxA*, *ribF*, *fur*, *gyrA*, *gryB*, *23S rRNA*, and *infB* genes. The authors found that the A2147G mutation of *23S rRNA* was significantly associated with resistance to clarithromycin (*p* = 0.00018), whereas resistance to levofloxacin was linked to D91N and N87K mutations of *gyrA*. Numerous mutations in the *rdxA* gene were also reported to be associated with resistance to metronidazole.

Taking these resistance genes into account, Liu and Nahata (contribution 4) reviewed treatments for *H. pylori* infection for patients with penicillin allergy. Because the prevalence of penicillin allergy ranges from 4% to 15%, the authors focused on treatments for patients with “true allergy,” specifically vonoprazan–clarithromycin–metronidazole and bismuth quadruple therapy, for which excellent cure and adherence rates have been reported. The authors suggested vonoprazan-based therapy as a possible first-line therapy but outlined that bismuth quadruple therapy could also be used if vonoprazan were to be unavailable. Levofloxacin- or sitafloxacin-based therapies were also noted to have moderately high efficacy, albeit with attendant serious side effects. The authors finally suggested the use of culture-based susceptibility testing to guide the selection of antibiotics.

Aligning with the previous study, Liu et al. (contribution 5) reviewed the efficacy and safety of vonoprazan and amoxicillin (VA) dual therapy for *H. pylori* infection. Their systematic review and network meta-analysis reported on the use of empiric therapies among treatment-naïve patients and showed that vonoprazan–amoxicillin–clarithromycin triple therapy was more effective than VA dual therapy. However, in terms of comparative safety, VA dual therapy ranked first.

Another review paper by Medakina et al. [3] assessed the molecular basis of antibiotic resistance in *H. pylori* and diagnostic methods with the aim of comprehensively examining the factors that mediate antibiotic resistance, the molecular mechanisms of resistance, and diagnostic methods used to evaluate resistance. Following their assessment, they attributed the emergence of resistance to unsuccessful eradication therapy regimens and the general use of macrolides and fluoroquinolones to treat other diseases, leading to the development of mutations in the *H. pylori* genes. The authors concluded that it was important to assess genetic determinants of *H. pylori* in combination with a diagnosis of resistance in order to curtail the pathogen’s further development of resistance to antibiotics, thereby increasing the effectiveness of anti-*H. pylori* therapies.

Similarly, a review by Jearth et al. discussed [4] drug-resistant *H. pylori* and its diagnosis using an evidence-based approach. Phenotypic and molecular methods of drug resistance were discussed in depth, and susceptibility-guided tailored therapy and empirical therapy of *H. pylori* were compared. The authors then concluded that the combination of whole-genome sequencing and traditional phenotyping would enable the discovery of novel resistances. However, they posited that an empirical therapy is the best method based on prior medication history. Additionally, the asserted that local resistance patters should guide the type of therapy chosen, particularly when finances are limited.

In their review of the global clinical implications of drug resistance in *H. pylori*, Argueta et al. (contribution 6) suggested that a paradigm shift to “tailored” (susceptibility-based) treatments, as opposed to empirical regimen selection, was needed to achieve improved clinical management of *H. pylori*. However, clinical trials have yet to show a clear superiority of such tailored treatments over empirical treatment regimens.

In another review article on the clinical implications of *H. pylori* resistance to antibiotics, Nista et al. (contribution 7) summarized data on antibiotic resistance in Italy (being the country in Europe with the highest antibiotic resistance rate) with the aim of finding the most efficacious approach for treating *H. pylori* infection. They confirmed increased resistance of the pathogen to clarithromycin, metronidazole, and levofloxacin. However, a satisfactory cure rate was observed when a bismuth-based regimen was used as first- or second-line treatment. Clarithromycin-based quadruple therapies resulted in good cure rates in naïve patients and could potentially be used as first-line treatment.

Waskito et al. (contribution 8) reviewed the opportunities and challenges in using metagenomic and metatranscriptomic approaches to assess antimicrobial resistance profiles in clinical practice. These two approaches were designed to overcome the limitations of the culture-based approach and are able to enhance the likelihood of detecting known resistance genes inside and outside the bacterial genome. Additionally, these techniques enable the detection of new resistance genes. However, their use poses intrinsic challenges in aspects such as cost and availability. Nonetheless, the timely usage of these approaches can improve the diagnosis made by clinicians and the management of these drug-resistant infectious diseases in humans.

Setshedi and Smith (contribution 9) reviewed various perspectives on antibiotic resistance and solutions for the effective management of *H. pylori* infections in Africa. Considering the paucity of data and unavailability of antimicrobial susceptibility testing, the authors suggested treating infected patients empirically, augmenting preventive measures, and involving stakeholders, such as medical and scientific professionals as well as the patients themselves, in order to develop a combined method to properly manage *H. pylori* infections in the African continent.

To facilitate screening for effective anti-*H. pylori* drugs, Mohamed et al. (contribution 10) developed a microtiter plate-based, bioluminescence-based drug screening method, which successfully identified the activities of fexinidazole and its metabolites against *H. pylori*. Fexinidazole and its sulfoxide and sulfone metabolites were found to be 3–6 times more potent than metronidazole. Therefore, in light of the recent FDA approval of fexinidazole and its good safety profile, this drug promises to be an effective alternative to metronidazole for treating *H. pylori* infection.

In their review, Gupta et al. [5] discussed innovative strategies that could improve *H. pylori* eradication, with a particular emphasis on directed delivery approaches. Examples of targeted delivery mechanisms included the gastro-retentive drug delivery system (GRDDS). These delivery systems were supposed to release medicine sustainably and stay in the stomach for long periods while providing copious amounts of drugs at the site of action. Other examples of targeted delivery were the use of nanoformulations of antibiotics, which is noteworthy for its decreased toxicity, increased bioavailability, and ability to provide controlled delivery. In conclusion, the authors recommended the eradication of *H. pylori* infection, a combination of nano-formulations of antibiotics integrated as a GRDDS.

Mosallam et al. [6] developed a nano-emulsion system that had the ability to carry curcumin and clarithromycin (Cur-CLR-NE) to protect patients against stomach acidity. Additionally, they increased the system’s efficacy against *H. pylori*. In vivo results from *H. pylori*-infected mice indicated that Cur-CLR-NE revealed a higher *H. pylori* clearance effect, in comparison to curcumin and clarithromycin using the same dose regimen and administration frequency with no damaging symptoms, compared to the positive control-infected mice.

Further discussing nanoparticles, Kamankesh et al. [7] reported on future nanotechnology-based strategies for the improved management of *H. pylori* infection. The authors posited a drug release paradigm that could prevent *H. pylori* antibiotic resistance, presenting a locally produced delivery system and engineered delivery platforms. These bioplatforms were noted to prevent development of antibiotic resistances and kill the *H. pylori* without exerting any attendant negative effects on the host microbiota.

Despite the fact that *H. pylori* has infected half of the world’s population and possesses an increasing resistance to drugs, there is still no available anti-*H. pylori* vaccine that can be used for prophylactic or therapeutic purposes. Therefore, Dieye et al. (contribution 11) have suggested strategies that could be developed to provide an effective DNA-based vaccine for *H. pylori.* Such a vaccine would have an oral recombinant vaccine delivery platform, be in its weakened form, have the ability to survive, be synthesized *in situ*, and be able to activate the appropriate components of the gut-associated mucosal immune system, among other processes.
